# Perceptual Biases in the Interpretation of Non-Rigid Shape Transformations from Motion

**DOI:** 10.3390/vision8030043

**Published:** 2024-07-04

**Authors:** Ryne Choi, Jacob Feldman, Manish Singh

**Affiliations:** Department of Psychology and Rutgers Center for Cognitive Science (RuCCS), Rutgers University, Piscataway, NJ 08854, USA

**Keywords:** shape from motion, structure from motion (SFM), non-rigid motion, biologically plausible transformations

## Abstract

Most existing research on the perception of 3D shape from motion has focused on rigidly moving objects. However, many natural objects deform non-rigidly, leading to image motion with no rigid interpretation. We investigated potential biases underlying the perception of non-rigid shape interpretations from motion. We presented observers with stimuli that were consistent with two qualitatively different interpretations. Observers were shown a two-part 3D object with the smaller part changing in length dynamically as the whole object rotated back and forth. In two experiments, we studied the misperception (i.e., perceptual reinterpretation) of the non-rigid length change to a part. In Experiment 1, observers misperceived this length change as a part orientation change (i.e., the smaller part was seen as articulating with respect to the larger part). In Experiment 2, the stimuli were similar, except the silhouette of the part was visible in the image. Here, the non-rigid length change was reinterpreted as a rigidly attached part with an “illusory” non-orthogonal horizontal angle relative to the larger part. We developed a model that incorporated this perceptual reinterpretation and could predict observer data. We propose that the visual system may be biased towards part-wise rigid interpretations of non-rigid motion, likely due to the ecological significance of movements of humans and other animals, which are generally constrained to move approximately part-wise rigidly. That is, not all non-rigid deformations are created equal: the visual systems’ prior expectations may bias the system to interpret motion in terms of biologically plausible shape transformations.

## 1. Introduction

One of the remarkable features of the human visual system is its ability to perceptually infer the 3D structure of an object from 2D motion cues in the projected image. Historically, research on the perception of shape from motion has been largely restricted to the perception of rigidly moving objects. This restriction stems from a widespread assumption in the field that the visual system needs to assume rigid motion in order to determine a 3D solution. Psychophysical studies on the perception of shape from motion have predominantly concerned the ability of observers to perceive the structure of rigidly moving objects.

To date, most of the work investigating the perception of shape from motion has been in the context of structure from motion (SFM). This research on SFM began with the study of the kinetic depth effect (KDE), a term coined by Wallach and O’Connell [1] to describe the spontaneous perception of a 3D solid object that occurs when an observer monocularly views the 2D deformation of a shadow projection of an object rotating rigidly in depth. (While the term “Kinetic Depth Effect” was coined by Wallach and O’Connell in [1], Metzger [2] discovered a similar phenomenon prior to their study. He found that the 2D shadow projections of vertical rods placed on a rotating flat disk are perceived as 3D rigid circular motion in depth [2].) In the study by Wallach and O’Connell, they projected the shadow of a 3D wireframe object onto a translucent screen. From a static view of the 2D shadow projection, there was no impression of a 3D shape. However, as the object started moving, observers were able to determine its 3D structure from the deforming 2D projection. The stimuli used in more modern SFM studies are usually quite different from the 3D connected-line-segment stimuli traditionally used in KDE studies (for a related shape-from-motion phenomenon that uses a distinct class of stimuli, see [3,4,5] on the stereokinetic effect). SFM studies usually use stimuli that consist of 3D objects with random dots sprinkled on their surface. Only the projection of the dots is shown to observers. While the stimuli may have changed, studies continued to focus on rigid motion. For example, Ullman [6] used a 3D cylinder with its surface covered in dots. From a single frame, only the 2D orthographic projection of dots covering the surface of an object is visible. Because the underlying shape is a cylinder, with its axis frontoparallel, the static frame appears as a rectangular region filled with random dots. As the cylinder rotates rigidly, observers perceive the 3D structure of the object. Numerous studies have found observers are good at perceiving the structure of a wide variety of complex 3D shapes from SFM [6,7,8,9].

The problem the visual system faces is underdetermined by the stimuli: for any 2D motion profile, there is an infinite number of possible 3D interpretations. The visual system must use constraints in order to specify a solution. Ullman [6] proposed rigidity as a constraint the visual system might use in SFM. (The distinction between rigid and non-rigid motions is, of course, an important one for the visual system. In shape from motion studies, observers do very well in discriminating between rigid and non-rigid motions [10,11]. Recent work has also shown that observers are sensitive to rigid vs. non-rigid motions in higher dimensions, such as 4D hypercubes [12].) Ullman was motivated by environmental plausibility: many of the objects in the environment move rigidly, and stationary objects in the world move rigidly in the projected image because of observer motion. He also proved mathematically that rigidity alone could uniquely determine a 3D interpretation under certain conditions (e.g., orthographic projection and opaque surfaces).

Ullman’s rigidity assumption states that if there is a unique interpretation of motion as a rigid body, then the visual system will take that interpretation. His SFM theorem incorporates the rigidity assumption and proves a theoretical minimum requirement of three views of four non-coplanar points [6]. Ullman later developed an incremental rigidity scheme to try to deal with small amounts of non-rigid motion [13]. That model could only handle small deviations from rigid motion from frame to frame and did not always converge on a stable solution [13,14].

Rigidity-based models have several important limitations. Rigidity was proposed as a constraint that “almost always holds true in the environment” [6]. However, many of the objects we encounter in the environment move non-rigidly, especially biological entities. Rigidity-based models are unable to interpret non-rigid motion. Moreover, some studies have shown that human observers have the ability to perceive non-rigid transformations based on image motion. Sinha and Poggio [15] demonstrated a compelling counterexample to the rigidity assumption. In their demonstration, they presented observers with rigid wireframe stick figures that resembled the human form. As the figure was rotated rigidly back and forth about its vertical axis, observers reported perceiving the figure as a human walking, hence changing its 3D shape non-rigidly. The rigidity assumption states that if a rigid interpretation is available, the visual system will take that interpretation; however, their demonstration presents a case where a rigid interpretation is available, but the visual system chooses a non-rigid interpretation. It is plausible, however, that their effect depended on the use of human stick figures, which could be readily recognized as such (indeed, their main aim was to investigate the role of learning in perception). Moreover, stick figures are different from the typical SFM stimuli defined by dots covering the surface of a 3D object. In the literature on biological motion (which is related to SFM but has been historically separated from it), observers have been shown to experience vivid, non-rigid perceptions from dots placed at the joints of humans and animals (rather than sprinkled all over their surfaces) [16]. Hence, the class of stimuli used in the biological-motion literature is quite different from those used in the SFM literature. Moreover, studies on biological motion demonstrate that a rigidity assumption is not necessary for correctly perceiving 3D shapes and motion. Even when no rigid interpretation is available, observers can still accurately perceive the structure of the object. However, it is possible that biological-motion stimuli engage a specialized module that is specifically tuned to recognize animate motion.

Within the context of SFM, a study by Jain and Zaidi [17] showed that people can be just as good at determining the structure of 3D objects undergoing certain non-rigid motions in SFM displays. In their study, they used curved 3D tubes/generalized cylinders where only the random dots placed on their surface were visible. They found that observers were just as sensitive to differences in the aspect ratio of the cross sections of tubes that were non-rigidly flexing in the image plane about the vertical and depth axes as they were to tubes that were rotating rigidly about those same axes. This study shows that human observers can determine 3D structure from objects moving non-rigidly in an SFM context. It again illustrates that the rigidity assumption is too restrictive to account for human perception. At least some non-rigid transformations, such as “flexing” of a single-part object, can be perceived by human observers [17].

**Figure 1:**
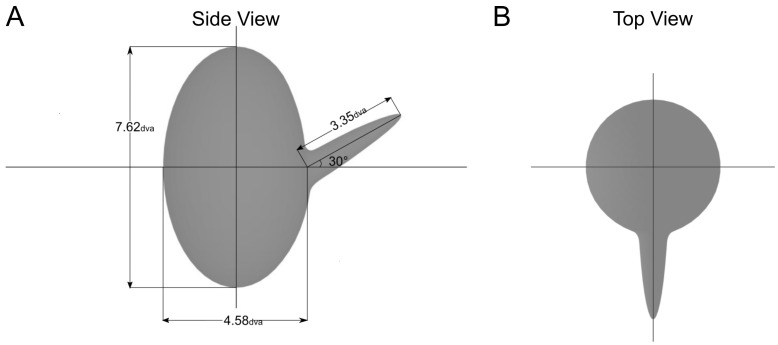
Cross section of the two-part 3D object used in Experiment 1. Dimensions of each part are shown in visual angles. The protruding part made a vertical angle of 30 degrees with respect to the main body. (**A**) Side view of the object. (**B**) Top view.

Our overarching goal is to explore the space of non-rigid transformations, specifically to understand what types of non-rigid 3D transformations are perceivable from image motion. (One can imagine that at extreme levels of non-rigidity, for example, with Brownian motion, no 3D structure would be perceivable. At the other extreme, observers are very good at perceiving the structure of objects undergoing rigid motions. However, between these two extremes, there are various levels of nonrigidity, many of which are perceivable.) To that end, we started by creating a two-part 3D object consisting of a main body with a smaller protruding part. We chose a two-part object because it can test part-wise non-rigidities (where the parts themselves are rigid, but the changing relationship between the parts makes the motion of the object as a whole non-rigid), as well as non-rigid transformations to one or both parts (see Figure 1 for the stimuli used in Experiment 1).

When we applied a non-rigid transformation to this object, we noted a surprising observation: a non-rigid part-length change was often perceptually reinterpreted as a change in the orientation of the part with respect to the main body. In the two experiments presented here, we studied this misperception that reveals the preferences and biases that the visual system brings to the interpretation of non-rigid shapes from motion.

To preview, the results of both experiments can be summarized as a bias for either part-wise rigid or rigid interpretations of non-rigid length change. We would like to emphasize, however, that part-wise rigidity is not simply a matter of ‘maximizing rigidity’. Previous work on shape-from-motion has emphasized a bias for rigidity, attempting to extend that bias into the realm of non-rigid transformations through incremental rigidity or minimal deviation from global rigidity [6,13]. This approach, however, treats all deviations from the global rigidity equally as noise. But, clearly, there are infinitely many ways that shape transformations can deviate from global rigidity, and they are treated very differently by the visual system. Thus, we emphasize that part-wise rigidity is not merely a matter of the visual system minimizing deviations from global rigidity. Rather, part-wise rigidity represents a distinct set of transformations within the infinite space of possible non-rigid transformations. We believe its special status for the visual system is a result of the environment in which our visual system evolved, where interpreting the motions of other humans and other animals was particularly important. The motion of these entities is approximated by mostly rigid parts articulating at joints with respect to each other [18]. Hence, if there is a possibility of interpreting a shape transformation in this way, it is preferred by the visual system. Part-length changes are comparatively rare. Although biological entities do grow their limbs over time, this does not typically occur on a time scale that is relevant for visual perception.

## 2. Experiments

To compare the perception of non-rigid part-orientation change and part-length change, we started by creating a two-part 3D object consisting of a large vertically oriented ellipsoid with a narrow half ellipsoid part protruding from its center. This object was defined by a random-dot texture on its surface.

The object was animated to rotate back and forth about its vertical axis while the protruding part underwent either an orientation change in the plane of the axis of rotation or a length change. When we developed the non-rigid length change stimuli, we noticed a misperception of non-rigid length change as a non-rigid orientation change. This tendency to interpret part-length change as part-orientation change is consistent with the idea that the visual system is biased toward part-wise rigid shape transformations. In the following two experiments, we used this two-part 3D object undergoing a non-rigid part length change to investigate this misperception further. In Experiment 1, our main goal was to document this misperception of non-rigid length change as a non-rigid orientation change. We used a yes/no task and seven levels of length change to explore the range of length change magnitudes that lead to a misperception of length change as an orientation change. We chose a yes/no task rather than a length vs. orientation change discrimination task because we worried that this would alert subjects to the possibility of multiple interpretations (i.e., they would be alerted to the fact that an orientation change was not the only way to perceive the stimuli). We should note, however, that our interest was not in measuring absolute thresholds for perceiving a change in the stimulus but rather in asking which type of change was more likely to be perceived; that is, comparing the relative proportions of perceived orientation change vs. length change for any given stimulus. Specifically, we were interested in whether there was a range of length-change magnitudes which would elicit reliably higher proportions of orientation-change responses than length-change responses. In Experiment 2, we again used seven levels of length change but used an adjustment task to explore a different misperception of length change in terms of part orientation. Both experiments explore how the visual system may be biased towards certain interpretations of non-rigid motion.

### 2.1. Experiment 1: Yes/No Task

#### 2.1.1. Method

*Subjects*

Five Rutgers University graduate students with normal, or corrected to normal, vision participated in the study. They were paid for their participation.

*Stimulus and Design*

The basic stimulus used in this experiment was a two-part 3D object. The object was a large ellipsoid with a protruding half-ellipsoid part. The object was drawn in the computer-assisted design software, OnShape, and exported as a .STL file to the animation software, Blender. In Blender, the object was animated and rendered with an orthographic projection. The larger ellipsoid body of the object was a 4.58° visual angle in width and had a height of 7.62° (Figure 1). The protruding part made a vertical angle of 30° with respect to the main body (the part was oriented 30° upward along the vertical plane) but had no horizontal angle (no orientation along the horizontal plane).

The two-part mesh object was covered in a random-dot texture. The dot texture was created by generating a grid of dots and randomly jittering the (x,y) positions of the dots. We used texture mapping to apply the image of the random dot texture to the 3D object. (Our stimuli are not pure “structure-from-motion” stimuli in the strict sense. Due to the texture mapping technique used to place the random-dot texture on the surface of the 3D object, our stimuli potentially include texture and contour cues. However, these cues are weak, as evidenced by the strong misperceptions of 3D shape and motion observed in both experiments.)

At the start of each motion sequence, the object was turned so that the protruding part was facing the observer (though it maintained an elevation angle of 30° throughout the sequence). The whole object rotated back and forth about its vertical axis ±25°. The object rotated to the right (observer’s right) 25°, then back to the center, then to the left 25°, then ended its motion when it rotated back to center. The object rotated at a constant speed throughout the motion. The temporal period was 3.96 s. Throughout the motion sequence, the protruding part never extended past the 2D projection of the larger body. Thus, the stimulus always appeared as an elliptical region filled with dots on every frame.

**Figure 2:**
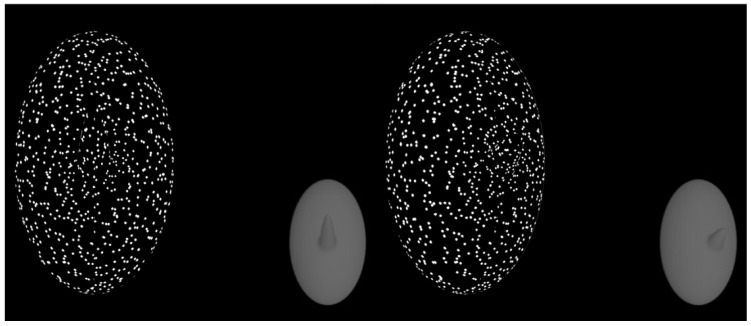
The pair of objects on the **left** shows the first frame of the stimulus when the part is front-facing. The textured object is what subjects actually see. The gray object in the bottom corner is the underlying 3D mesh object (subjects never see this). On the **right**, the object is rotated 25° to the right.

As the whole object rotated back and forth, the protruding part underwent a non-rigid length change. The part length was always the longest when the part was facing the observer. As the object rotated away from the observer, the part would decrease in length. As the object rotated back to its original position (facing the observer), the part non-rigidly increased in length back to its original length. The degree of length change was manipulated. Figure 2 shows the textured object and its underlying 3D surface when the object was front-facing (left) and when the object was rotated 25° (right).

Seven levels of length change were used in this experiment. In this experiment, we refer to the length changes by length-change ratio. The length-change ratio is the ratio of the part length at its longest (the starting length when the part is front-facing) to its shortest length (length when the whole object is rotated to ±25°). The starting length of the part was always fixed across all levels of length change. The seven levels of length ratio were 1.1976, 1.2903, 1.3986, 1.5267, 1.6807, 1.8692, and 2.1053. The experiment was divided into two blocks. In each block, each level of length ratio was shown 20 times for a total of 140 trials per block and 280 trials total.

*Procedure*

The experiment was run in a dark room. Subjects were seated 75 cm from an iMac monitor with a refresh rate of 100 Hz and a screen dimension of 800 × 1280 pixels. The experiment was programmed and run in MATLAB using the Psychtoolbox library [19].

For every stimulus, we wanted to ask two questions: one about length change and one about orientation change. However, we chose not to ask both questions together after each stimulus presentation because of concerns that (1) this may alert subjects to the possibility of multiple interpretations, and (2) this may make their two responses mutually dependent (e.g., if the subjects felt that their two responses need to be mutually consistent). Therefore, we decided to run two blocks showing the same stimuli but asking different questions to encourage independence between the two responses. Instructions were presented before the start of each block. In the first block, subjects were instructed to make a judgment about the protruding part and to respond “yes” or “no” to the following question: did you see an orientation change (i.e., a change in the orientation of the protruding part)? Subjects were then shown two examples of orientation change. In these examples, a two-part object made of a cylinder body with a protruding cylinder part underwent a non-rigid orientation change. The protruding cylinder part changed its vertical orientation up and down. In the example demos, the whole object did not rotate back and forth, and the object had a shaded (non-textured) surface. The two examples showed the same non-rigid orientation change but from different viewing angles.

Before the start of the second block, subjects were instructed to make a judgment about the protruding part and to respond yes or no to the following question: did you see a length change (i.e., a change in the length of the protruding part)? Subjects were then shown two examples of length change. The same two-part cylindrical object was used in these example stimuli. However, in these examples, the part had no vertical or horizontal orientation with respect to the body (the part was perfectly perpendicular to the body), and the part non-rigidly increased and decreased in length.

In both blocks, after watching the two example demos, subjects could press any key to move on to the experimental trials. At the beginning of each trial, one of the seven possible length change ratio stimuli described above was presented. The stimulus was then removed from the screen, and a question screen appeared asking in the first block: "Did you see an orientation change (i.e., a change in the orientation of the protruding part)?" and in the second block: "Did you see a length change (i.e., a change in the length of the protruding part)?". Subjects used the right arrow key to respond ‘yes’ and the left arrow key to respond ‘no’. No time limit to respond was imposed. Subjects could take a break between blocks. In each block, the order of the seven different length ratio stimuli was randomized. It took approximately 40 min for subjects to complete the experiment.

#### 2.1.2. Results and Discussion

**Figure 3:**
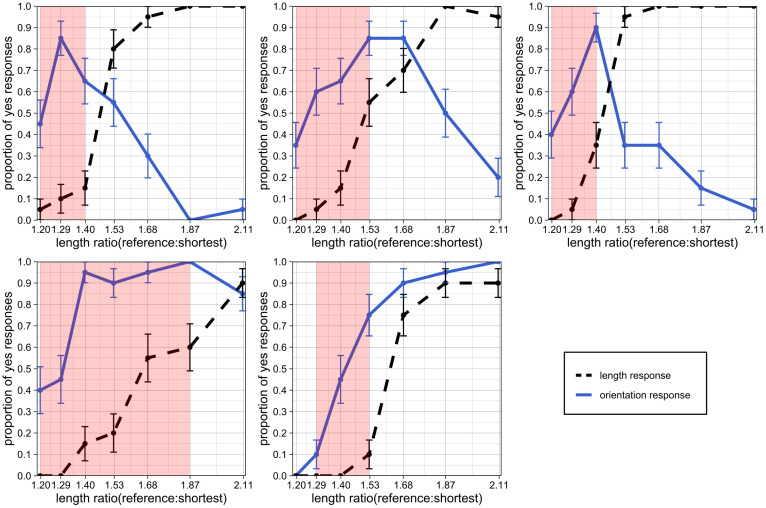
Proportion of “yes” responses to the prompt “Did you see an orientation change?” (plotted as a blue solid line) and to the prompt “Did you see a length change?” (plotted as a dashed black line) as a function of increasing length ratio (magnitude of length change). The five plots show data from individual observers 01–05. Error bars represent standard error. The red regions represent ranges where the orientation-change response was statistically higher than the length-change response, thereby indicating a perceptual reinterpretation of length change as orientation change.

The graphs in Figure 3 plot the proportion of ‘yes’ responses as a function of length ratio. Each plot represents data from one subject. The solid blue line represents the proportion of ‘yes’ responses submitted by subjects in the first block to the question “Did you see an orientation change (i.e., a change in the orientation of the part)?”. The dashed black line represents the proportion of ‘yes’ responses submitted by subjects in the second block to the question “Did you see a length change (i.e., a change in the length of the part)?”. The error bars represent standard error.

The length response for each subject, represented by the dashed black line, is highly consistent across observers. As the degree of length change (length ratio) increases, the proportion of ‘yes’ responses to the question “Did you see a length change?” increases monotonically.

The orientation response, represented by the solid blue line, is less consistent across subjects. However, the difference among observers occurs primarily at the highest levels of length change, and there seem to be two response patterns. For some subjects, the “illusion” of length change as orientation changes seems to break down at the highest levels of length change (hence, the curve goes down after reaching a peak). For other subjects, the illusion remains, and they continue to perceive an orientation change no matter how large the length change becomes.

**Figure 4:**
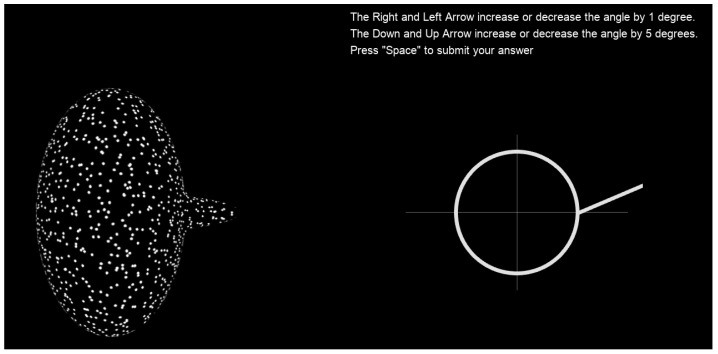
Subjects matched the perceived horizontal orientation of the part with respect to the main body in the random-dot display on the left using the adjustment display on the right. The adjustment display is a schematic of the top view of the 3D object. The stimulus remained on display during the whole trial. Adjustment instructions remained above the adjustment display.

For each subject, there is a clear range of length ratio values where the proportion of ‘yes’ responses to the question "Did you see an orientation change?" is significantly higher than the proportion of ‘yes’ responses to the question "Did you see a length change?". The red-shaded regions on the plot in Figure 4 represent regions where the proportion of ‘yes’ responses in the first block is higher than the proportion of ‘yes’ responses in the second block and where the standard errors do not overlap. We observe that even at substantial levels of length change, some subjects reported seeing an orientation change instead.

### 2.2. Experiment 2: Horizontal Angle Adjustment

#### 2.2.1. Method

*Subjects*

Five Rutgers University graduate students with normal, or corrected-to-normal, vision participated in the study. They were paid for their participation.

*Stimulus and Design*

The same type of two-part 3D objects used in the first experiment was used for this second experiment. The same dot texture used in the first experiment was also used for these stimuli. However, there were several key differences in the stimuli used in the second experiment. First, in this experiment, the protruding part did not point upward with respect to the main body. The protruding part was perpendicular to the body in both the vertical and horizontal directions. Second, the object did not start off with the protruded part facing the subject. Instead, the object was oriented 45° degrees to the right, facing away from the subject. Third, the whole object rotated back and forth about the vertical axis of the large ellipsoid between 45° and 90°. When the object was rotated 90°, the part was fully visible in the 2D projected silhouette.

The second experiment also included stimuli where the length of the part would increase non-rigidly. In the first experiment, the length of the part always decreased as the whole object rotated away from its starting position (front-facing) and increased as it came back to the front. This experiment also had stimuli where the length of the protruding part non-rigidly increased as the whole object rotated away from its starting position (at 45°) until the end of its rotation at (90°). Then, as the whole object rotated back to its starting position, the length of the protruding part decreased back to its original length.

There were seven levels of the degree of length change, i.e., non-rigid length changes in the protruding part. In this experiment, we took the length ratio to be the ratio of the length of the part at 90° (peak of whole body rotation) to the length of the part at 45° (starting point). So, in length-increasing conditions, the ratios were greater than 1, whereas in length-decreasing conditions, the ratios were less than 1. We had three levels of increasing length where the part non-rigidly increased to 1.24,1.48, or 1.72 times its original length. There was one level of no length change, where the length ratio was 1. Furthermore, there were three levels of decreasing length where the part non-rigidly decreased to $\frac{1}{1.24},\frac{1}{1.48}$, and $\frac{1}{1.72}$ times its original length. Each of the seven levels was presented ten times each for a total of 70 trials.

To report the perceived horizontal angle, subjects were asked to make adjustments to a schematic figure. The figure, as seen on the right side of Figure 4, was a white circle with a protruding white line part. The schematic represents the view of the 3D object from the top: the circle represents the main body, while the line represents the protruding part. The diameter of the circle was 2.14°, while the line was 2.35° long. Subjects adjusted the orientation of the white line in the adjustment display to match the perceived horizontal orientation of the part relative to the main body in the random-dot display (see below for details).

*Procedure*

The experiment was run in a dark room. Subjects were seated 75 cm from an iMac monitor with a refresh rate of 100 Hz and a screen dimension of 800 × 1280 pixels. The experiment was programmed and run in MATLAB using the PsychToolbox library [19].

Instructions were presented at the start of the experiment. Subjects were asked to make a judgment about the horizontal orientation of the protruding part with respect to the main body. They were asked to adjust the schematic figure on the right of the screen to match their perception of the horizontal angle. The arrow keys were used to adjust the angle of the line (with respect to the circle) on the schematic. It was explained to the subjects that the schematic represented a top-down (looking down on) view of the object. Adjusting the line downward meant that the part was perceived to be angled toward the observer, and adjusting the line upward meant that the part was perceived to be angled away from the observer. The angle could be adjusted in increments of either 1° or 5° (using two different sets of keys). The 1° increments allowed subjects to make finer adjustments once their setting was in the right ballpark.

At the bottom of the instructions screen were five examples of two-part 3D objects with different horizontal angles between the protruding part and the body. The two-part objects were cylinders with protruding cylinder parts. They were not defined by a random-dot texture but were given a wood-grain texture with shading. Each figure was oriented 45 degrees away from the observer. From left to right, the parts had a horizontal angle of −40°, −20°, 0°, 20°, and 40°. Above each 3D figure was a schematic showing the “correct answer” to each of the oriented 3D objects. Part of the goal of these examples was to make it clear to the subject that the relevant angle they were meant to match was the (horizontal) orientation of the part relative to the main body of the object and not the orientation of the part in space with respect to the observer. Once subjects understood the task, they could press any key to continue with the practice trials. There were seven practice trials. For each trial, one of the seven levels of length ratio was shown in a random order. Once the practice trials were over, the subjects continued to the real trials.

During each trial, the random-dot stimulus was continuously displayed on the left side of the screen while subjects made their angle adjustments to the schematic on the right side of the screen. Once they felt their adjustment of the schematic matched their perception of the orientation of the protruding part with respect to the main body of the 3D object, they submitted their response by pressing the space button and moved on to the next trial. At the end of each trial, the adjusted angle response was collected. There was no time limit on each trial. Seventy trials were presented in a random order for each subject. The experiment took approximately 25 min.

#### 2.2.2. Model Predictions

**Figure 5:**
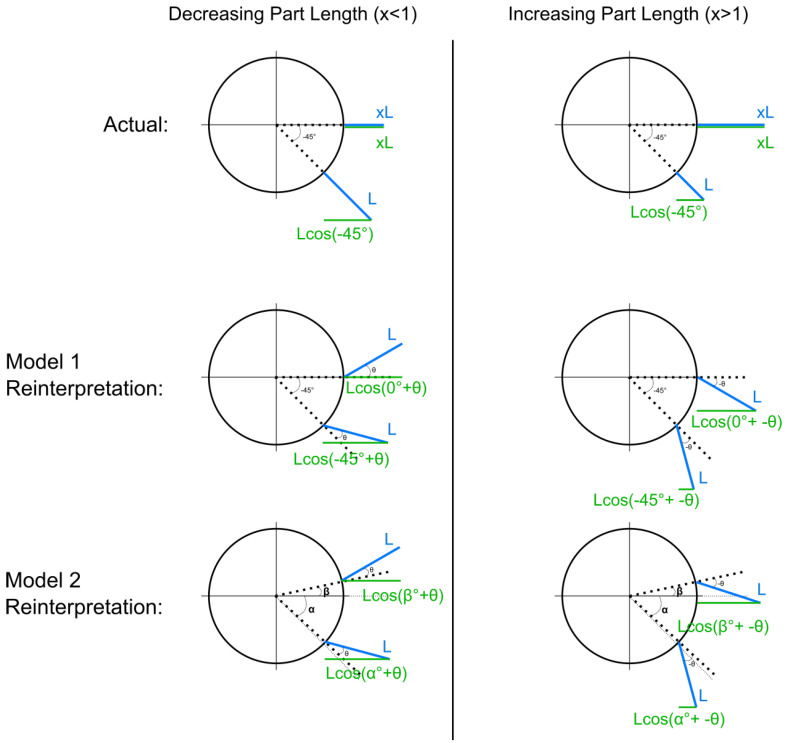
The top row represents the actual length change in the figure as the part goes from −45° to 0°. The column on the left is the decreasing case, and the right column is the increasing case. Blue lines represent the part, and green lines represent the part’s projection on the image plane. The middle row represents Model 1’s geometric reinterpretation of the length change as a part with a fixed horizontal angle and no length change. The bottom row represents Model 2’s reinterpretation of length change based on a version of the geometric model that does not assume that subjects accurately perceive the start and end orientation of the object and instead leaves the start and end orientation as free parameters (α and β, respectively).

We created two models based on the assumption that length change is being reinterpreted as a fixed horizontal angle with no length change. Under these assumptions, the models make predictions of the perceived horizontal angle using the magnitude and direction of length change. Figure 5 illustrates our models.

The top row of the figure represents what is “actually” going on in our stimuli. The blue lines represent the part. On the left side of the figure are schematic illustrations of the length-decreasing case. The part length goes from L, its original length, to x·L (decreasing so x<1) from −45° to 0°. On the right side of the diagram, the part is increasing to *x* times its length from −45° to 0°.

#### 2.2.3. Model 1: Geometric Model

The two schematics in the middle row of Figure 5 show the projected lengths of the part under the reinterpretation of the part having a fixed horizontal angle with no length change. In both schematics, the length of the part (represented by the blue line) stays fixed from −45° to 0°. On the left, in the decreasing-length case, the part is perceived to be angled away from the observer. We use θ to represent the perceived part angle. The solid green lines represent the part’s projected lengths in the image plane. The projected length changes in the image plane for the actual length change (the two schematics in the top row) and the projected length changes in the image plane for the reinterpretation (middle row) must be equated. The ratios between the lengths of the parts in the image plane from start to end must be the same for both “what is really happening” (top row) and for the “reinterpretation” (middle row). So, we equate the ratios and obtain the following equations:

$$\frac{xLcos(0^{\circ })}{Lcos(-45^{\circ })}=\frac{Lcos(0^{\circ }+\theta )}{Lcos(-45^{\circ }+\theta )},$$

$$x=\frac{cos(\theta )}{cos(\theta )+sin(\theta )},$$

$$x=\frac{1}{1+tan(\theta )},$$

(1) $$\theta =tan^{-1}\left(\frac{1}{x}-1\right).$$

**Figure 6:**
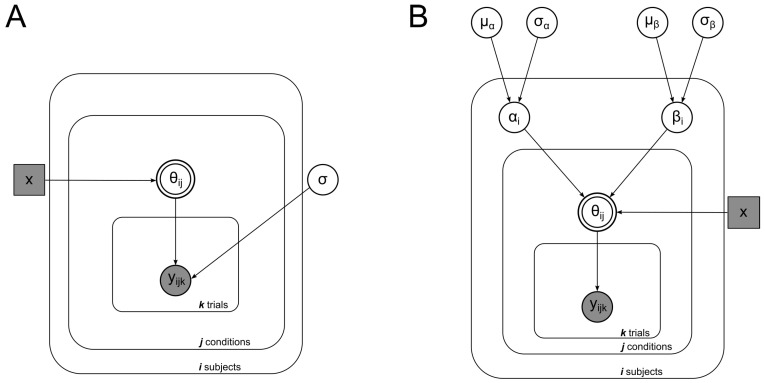
Bayesian graphical models illustrating the structure of Model 1 (left) and Model 2 (right). The basic structure is the same for both models: data $y_{ijk}$ for each subject *i*, in condition *j*, and on each trial *k*, are drawn from a Gaussian distribution centered around θ, which is a deterministic function of *x*, the magnitude of length change. (**A**) Model 1 essentially models subject response centered around a deterministic function (Equation (1)) with noise. (**B**) In Model 2, responses are centered around Equation (2), which added as free parameters, α and β, which represent the perceived start and end orientation of the object.

In the decreasing case, $x=\frac{1}{1.72},\frac{1}{1.48}$, or $\frac{1}{1.24}$. In the increasing case, x=1.24,1.48, or 1.72. In the no-length-change case, x=1. Solving θ, we input the seven levels of length ratio used in our experiment to obtain predictions of the perceived part angle. This deterministic geometric model, with no free parameter, was expanded with a probabilistic assumption in order to model subject data and perform model comparisons. We modeled subject responses to each trial and each condition to be from a Gaussian distribution centered on the value of θ given by the deterministic equation (Equation (1)) and with a standard deviation that we inferred from the data. Figure 6A is a graphical representation of this model.

#### 2.2.4. Model 2: Geometric Model with Orientation Uncertainty

The geometric model described above assumes that subjects perfectly perceive the start and end orientation of the object. That is, the object rotates from −45° to 0°. We relax the assumption that subjects perfectly perceive the start and end orientation and allow those values to be free parameters representing α (start orientation) and β (end orientation). Substituting α for −45° and β for 0°, we obtain the following new equations:

$$\frac{xLcos(0^{\circ })}{Lcos(-45^{\circ })}=\frac{Lcos(\beta +\theta )}{Lcos(\alpha +\theta )},$$

$$\sqrt{2}x=\frac{cos(\beta )-sin(\beta )tan(\theta )}{cos(\alpha )-sin(\alpha )tan(\theta )},$$

(2) $$\theta =tan^{-1}\left(\frac{\sqrt{2}\cdot xcos\left(\alpha \right)-cos\left(\beta \right)}{\sqrt{2}xsin\left(\alpha \right)-sin\left(\beta \right)}\right).$$

To infer each subject’s perceived start (α) and end (β) orientation in this model, we created the Bayesian hierarchical model illustrated in Figure 6B. We assumed that data are generated from a Gaussian distribution centered around the value given by Equation (2) for θ. Equation (2) takes α and β as free parameters. As these variables represent the start and end orientation of the object, we assume that they come from Gaussian distributions centered around the true values of the start (−45°) and end (0°) orientations. From this model, we infer the values of α and β.

#### 2.2.5. Model Comparison

We performed a model comparison between Model 1 and Model 2. We used MCMC samples (from JAGS) to calculate the deviance information criterion (DIC), which is a measure of goodness-of-fit that includes a penalty for models that contain more parameters [20]. We calculated the difference in DIC between the probabilistic model ($DIC_{M2}=2902$) and the geometric model $\left(DIC_{M1}=3610\right)$. We found a difference of $\left(DIC_{M2}-DIC_{M1}=-707.8\right)$, which is strongly in favor of the probabilistic model.

### 2.3. Results and Discussion

**Figure 7:**
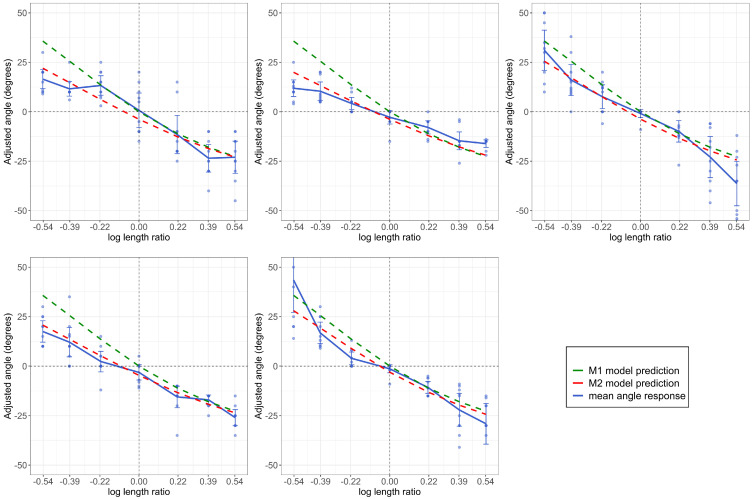
Each plot represents a different subject. The adjusted angle response is plotted as a function of the log length ratio. Each point represents a response to a trial. The mean of the angle responses is plotted as a blue solid line, while the model prediction is plotted as a dashed green line. The vertical reference line separates the decreasing length ratio (negative) from the increasing one. The horizontal reference line separates upward angle adjustments (negative) from downward (positive) ones.

In Figure 7, we plotted mean angle settings as a function of the log length ratio. Each subplot shows the data from one observer. On the x-axis, the negative values represent the decreasing length cases (the length ratios were $\frac{1}{1.24},\frac{1}{1.48}$, and $\frac{1}{1.72}$) and the positive values represent length-increasing conditions. For the mean angle responses, the positive values mean the subjects adjusted the angle of the white line upward from horizontal (i.e., the part was seen as angled away from the observer). The negative values mean the subjects adjusted the angle of the white line downward from horizontal (i.e., seen as angled towards the observer). The error bars are 95% confidence intervals. The dashed green line represents our Model 1 prediction; that is, the values of θ given by Equation (1) for each level of the length ratio. The dashed red lines are the predictions of Model 2. The predictions of Model 2 are different for each observer. We first took the mean of the sampled posterior distributions of α and β for each subject. Then, for each subject, we took those (fixed) values and plugged them into Equation (2) to calculate the predictions of the model.

Each subject perceived some “illusion” of the horizontal part angle when there was none (recall that the part was always perpendicular to the main body in all cases, which corresponds to a horizontal angle of 0° on this scale). Although veridical perception would lead to horizontal angle settings of 0° for each level of length ratio, we can see that subjects consistently reported perceiving a horizontal angle. Subjects also perceived the horizontal angle in the direction that was predicted. In the decreasing part length cases (negative log length ratio), subjects adjusted the angle in the adjustment display upward (positive mean-adjusted angle response), meaning they perceived the part to be pointing away from them. In length-increasing conditions, subjects adjusted the angle in the adjustment display downward, meaning they perceived the part to be pointing toward them. This was consistent for all subjects.

Given that Model 1 has no free parameters and thus involves no “fitting” to the data, it does surprisingly well in predicting the subject’s angle settings. Recall that both models are based on the single assumption of reinterpreting length change as a (fixed) horizontal angle with no length change. Although some deviations are apparent for extreme negative values, the overall fit of the model suggests that this single assumption goes a long way in explaining observers’ perception of part orientation in these displays. Additionally, Model 2 provided an interesting insight into how each subject perceived the start and end orientation of the object. The perception of start orientation was near the ground truth value (−45°) for each subject (mean of the posteriors of α for each subject ranged between −43.71° and −41.89°); the perceived value of the end orientation was much more varied for each subject (mean of the posteriors of β ranged from 9.16°–19.35°) and further from the ground truth (0°).

## 3. General Discussion

The literature on perception of shape from motion has primarily focused on rigid body motion. Most of the models and psychophysical investigations have either implicitly assumed rigidity or at least stayed within the boundaries of investigating the ability to perceive the structure of rigidly moving objects. While the assumption of rigidity is useful and environmentally plausible for many objects, focusing solely on rigidity leaves out an enormous set of possible non-rigid motions. As we have mentioned, many objects in the environment move non-rigidly, especially biological entities. Indeed, in light of the prevalence and salience of biological objects in our environment, a model that excludes non-rigid objects cannot claim to be “ecological”. We have seen from demonstrations like those by Sinha and Poggio [15] and Jain and Zaidi [17], that the rigidity assumption is too restrictive.

The experiments presented here are motivated by an overarching goal to begin exploring the space of non-rigid transformations that can be perceived through the inference of shape from motion. In both experiments, we focused on exploring our initial observation that when our two-part 3D object stimulus underwent a non-rigid length change to its smaller protruding part, it was often perceptually reinterpreted as undergoing a different type of non-rigid transformation. We explored these misperceptions to gain insight into the biases the visual system brings to the interpretation of non-rigid shapes from motion.

In Experiment 1, we observed that a non-rigid length change was reinterpreted as a non-rigid part orientation change. We were able to show a clear range of length-change values where this misperception or bias towards an interpretation of part-orientation change existed for each of our subjects. In Experiment 2, where we made the non-rigid length change fully visible in the silhouette, we observed the non-rigid length change reinterpreted as a part with no length change but an illusory horizontal angle. In this experiment, we not only observed a range of length-change values where a misperception occurred but also produced parametric data that could be compared against the predictions of a simple geometric model that reinterprets length change in terms of a fixed horizontal angle between the part and the body with no length change. The predictions of the model closely matched the orientation settings of subjects, suggesting that their visual systems may be making similar assumptions. In both experiments, we observed a clear bias against length change interpretation in favor of an interpretation involving either a changing part orientation (in Exp. 1) or a fixed but “illusory” part orientation (in Exp. 2) between the part and the main body. We believe that the findings from the two experiments presented here suggest that the visual system may be biased towards part-wise rigid interpretations of non-rigid transformations. This may be because part-wise rigid interpretations are more biologically plausible. Objects in the environment that move non-rigidly are typically biological entities, especially humans and other mammals. The motions of these entities are usually constrained to certain types of non-rigidities that can be defined largely in terms of part-orientation change (i.e., the articulation of limbs, such as the raising of an arm, is a non-rigid part orientation change). Such shape transformations are also particularly well captured by skeleton-based representations of shapes [21,22]. Previous work has shown that biological plausibility influences the perception of apparent motion [23] and sensitivity to shape change [24]. In our study, we see this influence on the perception of non-rigid 3D shape transformations from motion.

## 4. Conclusions

The visual system has the remarkable ability to perceive the 3D structure of objects from 2D image motion. Research on the perception of shape from motion has been ongoing for a long time but has largely restricted itself to the domain of rigid motion. However, it is clear that some forms of non-rigid motion are readily perceivable when inferring shape from motion. Our overarching goal is to understand what types of non-rigid transformations are more readily perceivable. The current study suggests that the distinction between biologically plausible and implausible shape transformations may be an important factor in addressing this larger question. In the two experiments presented here, observers saw part length change perceptually reinterpreted as more biologically plausible transformations. In Experiment 1, part length change was perceptually reinterpreted as part orientation change. In Experiment 2, with a slightly different geometry and viewing angle of the object, a new interpretation emerged, this time of a rigidly attached part with an illusory horizontal angle relative to the main body. Biologically plausible motions, characterized by a certain set of non-rigid shape transformations, likely hold special significance for the visual system, which has evolved a bias towards such motions over time due to its frequent exposure to and interest in the movements of humans and animals.

## Data Availability

The data are available upon request from the corresponding author.
